# Supplementary figures and images for: Omitting age-dependent mosquito mortality in malaria models underestimates the effectiveness of insecticide-treated nets
Source: PLoS Comput Biol. 2022 Sep 19;18(9):e1009540. doi: 10.1371/journal.pcbi.1009540 (PMC9522293; doi:10.1371/journal.pcbi.1009540)

Control

Treated

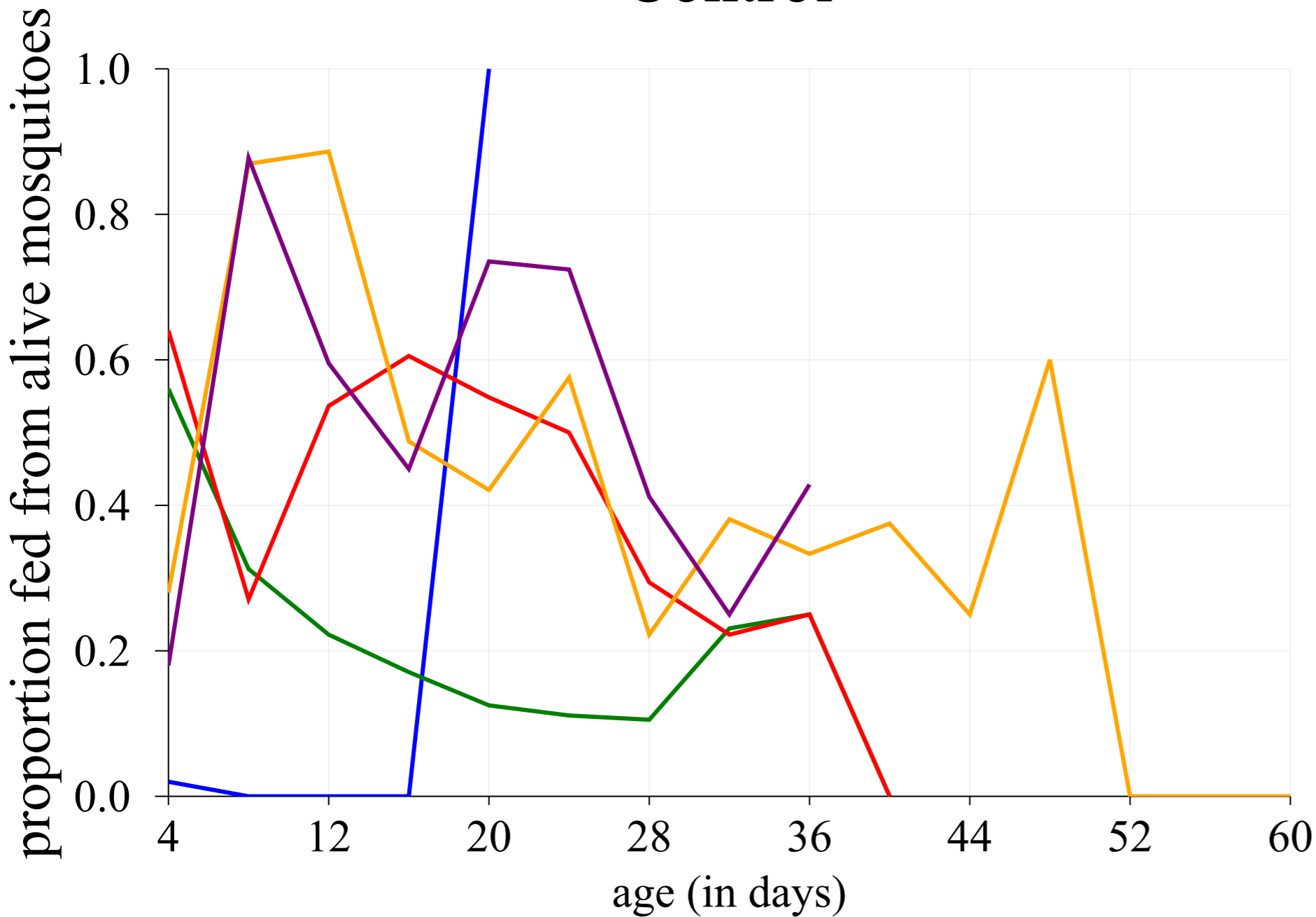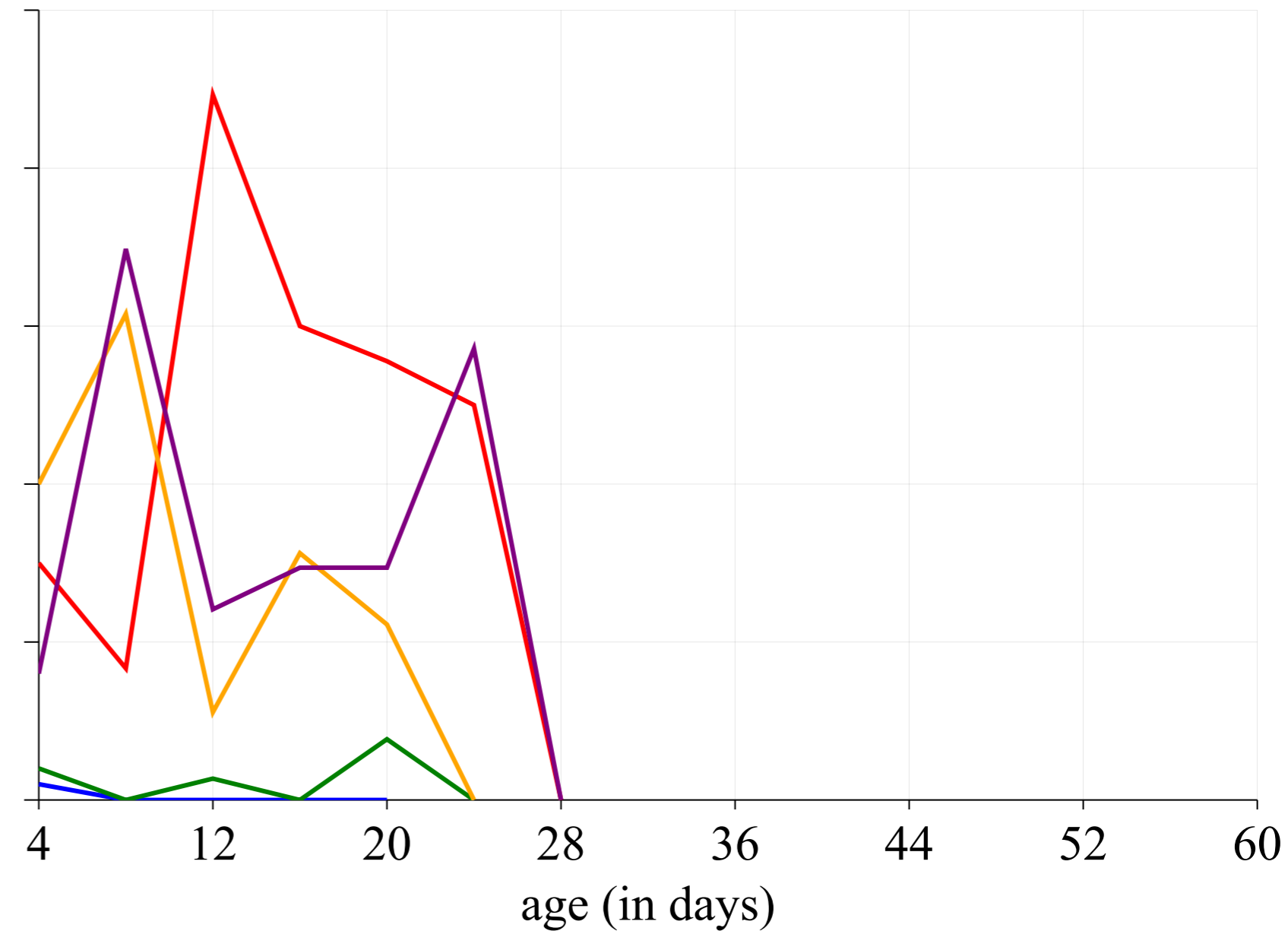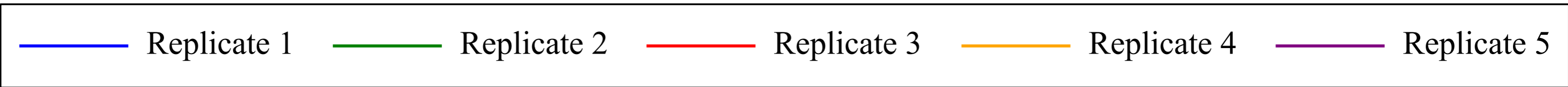

Supplement: S1 Fig — The results from Replicate 1 are a lot different than the rest of the replicates. On the control plot (left), even at the beginning where many mosquitoes are still alive, the proportion of those that were fed is (close to) zero, and only at the very end a mosquito actually feeds. Similarly, on the right, almost all of the mosquitoes go through their lifetime without feeding. (PDF) [file pcbi.1009540.s009.pdf]

Control

Treated

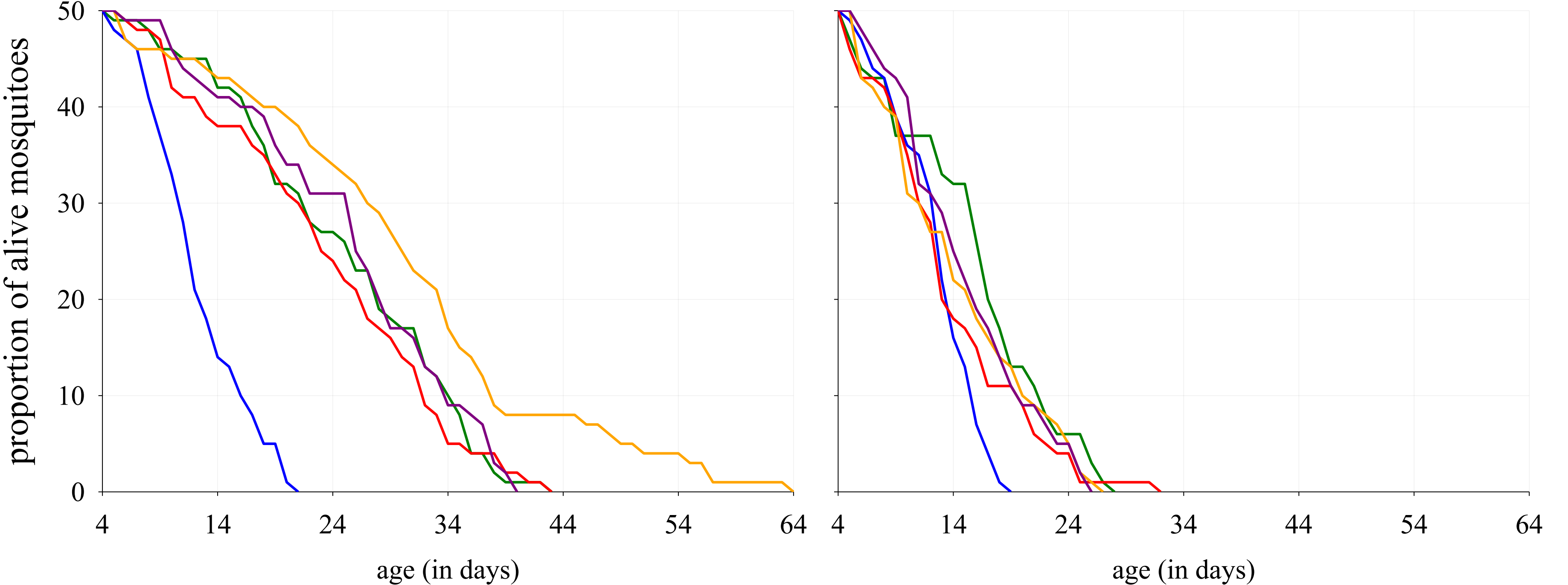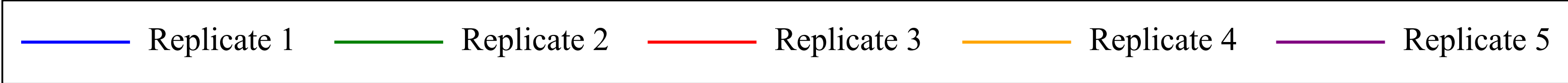

Supplement: S2 Fig — In both plots Replicate 1 seems different to the trend followed by the other replicates, especially for the control case (left). This could be explained by the trends in S1 Fig. (PDF) [file pcbi.1009540.s010.pdf]

## Age-Independent

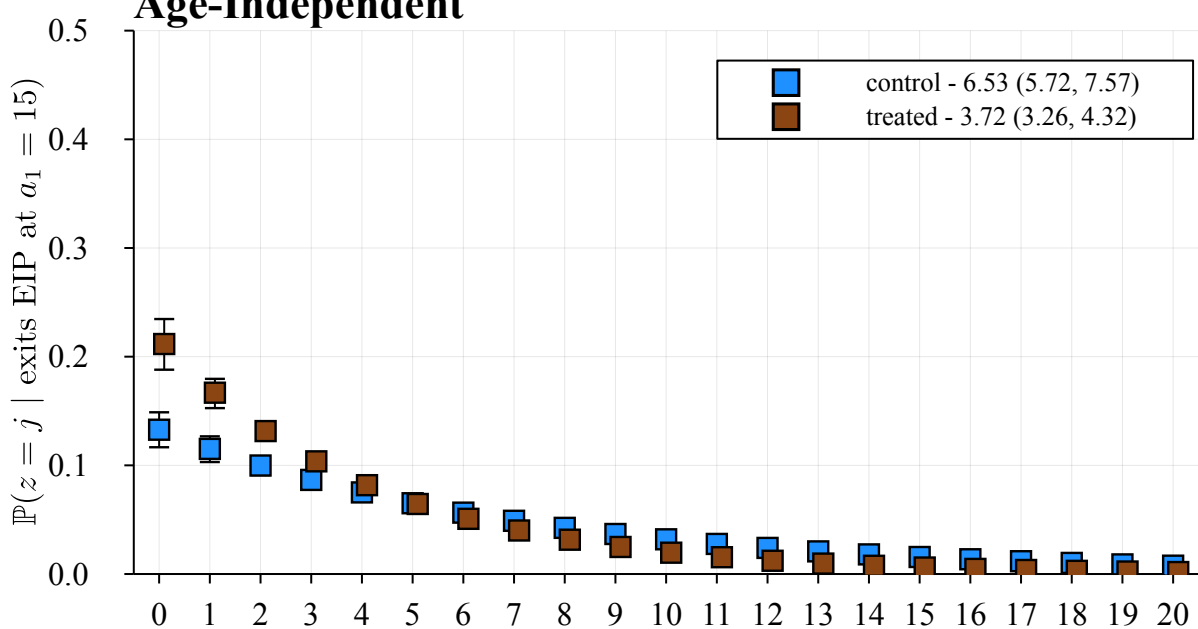

## Logistic

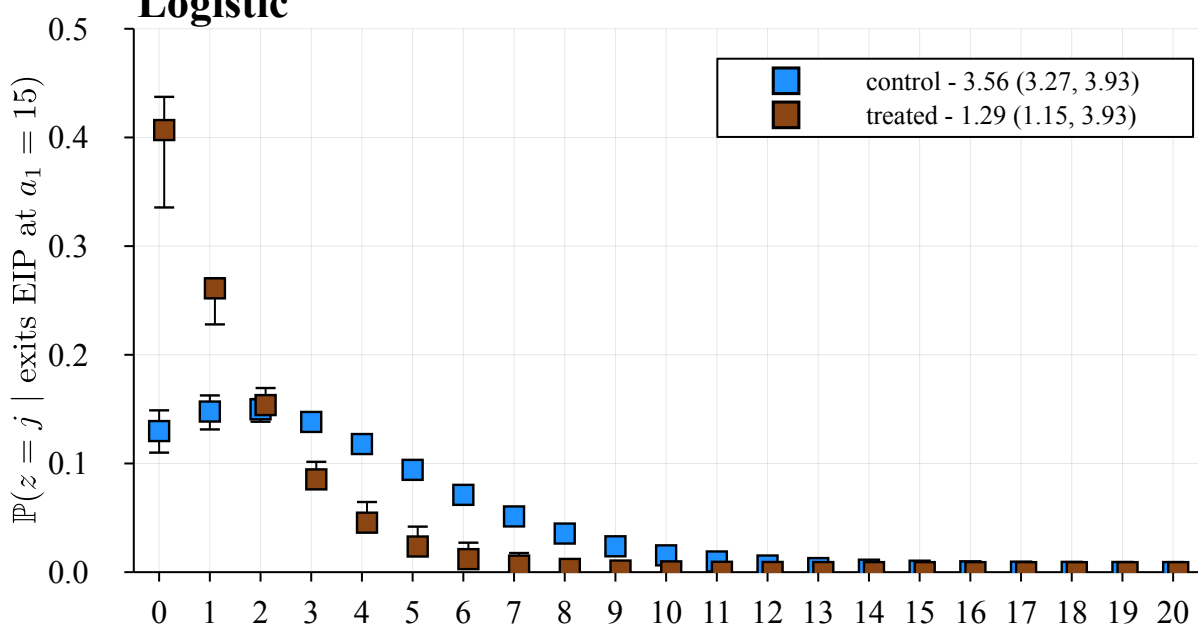

## Gompertz

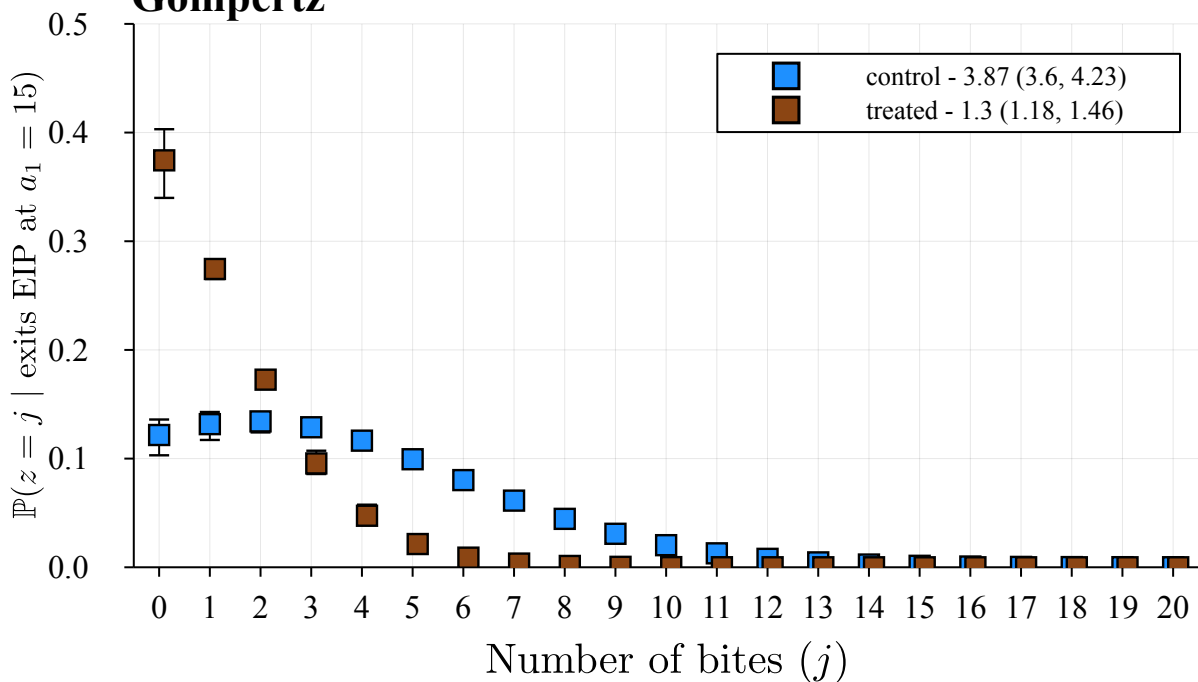

Supplement: S3 Fig — The average number of bites for each treatment is found in the legend box of each plot. The error bars represent the propagated uncertainty of the estimated parameters. We can see that the highest probabilities are for the smaller values of j in all cases. The probability the number of bites is closer to zero is higher in the treated than in the control cases. The average number of bites is higher for the control treatment for all three cases, as expected. We also notice that for the treated case and the age-dependent functions the probability that the number of bites is equal to anything above seven is essentially zero. However, for the age-independent graph this probability goes to zero for a much higher value of j, which again shows how unrealistic an age-independent assumption is. (PDF) [file pcbi.1009540.s011.pdf]
